# Finding Meaning Amidst COVID-19: An Existential Positive Psychology Model of Suffering

**DOI:** 10.3389/fpsyg.2021.641747

**Published:** 2021-03-10

**Authors:** Daryl R. Van Tongeren, Sara A. Showalter Van Tongeren

**Affiliations:** ^1^Department of Psychology, Hope College, Holland, MI, United States; ^2^Private Practice, Holland, MI, United States

**Keywords:** positive psychology, existential psychology, suffering, COVID-19, positive psychology (PP2.0)

## Abstract

The global COVID-19 pandemic has created a crisis of suffering. We conceptualize suffering as a deeply existential issue that fundamentally changes people indelible ways and for which there are no easy solutions. To better understand its effects and how people can flourish in the midst of this crisis, we formally introduce and elaborate on an Existential Positive Psychology Model of Suffering (EPPMS) and apply that to the COVID-19 global pandemic. Our model has three core propositions: (a) suffering reveals existential concerns, (b) existential anxiety impairs one's ability to find meaning, and (c) cultivating meaning is the primary way to address suffering and allay existential anxiety, eventually leading to flourishing (and potentially growth). We apply this model to the COVID-19 pandemic, including how to build meaning, and discuss clinical implications.

## Introduction

The global COVID-19 pandemic has created a crisis of suffering. As of this writing (January, 2021), the virus has infected more than 100 million people worldwide, claiming the lives of more than 2 million, and disrupting the lives of people in nearly every country on the planet (Johns, 2021). Economies have halted. Schools have been shifted to remote learning. Businesses have closed. Daily routines have been significantly altered. Social isolation is rampant. Life has changed in dramatic ways. We do not think it is an understatement to say that this pandemic will leave an indelible mark on this generation of humanity. So, then, how to do people cope with suffering on this scale—suffering that has revealed core existential fears and altered their lives in profound ways?

In this paper, we propose a new theoretical approach to suffering and apply this to the COVID-19 pandemic. First, we define suffering as an existential issue. We then explicate the core tenets of our new model and highlight how it can explain responses to the global pandemic. Next, we develop the clinical implications of our model, and we discuss avenues for advancing research in this domain. Finally, we discuss unanswered questions and suggestions for future inquiry.

## The Existential Positive Psychology Model of Suffering (EPPMS)

We propose an Existential Positive Psychology Model of Suffering (EPPMS), which is informed by, and lies at the intersection of, both existential psychology—a perspective that addresses core fundamental questions about what it means to be human (Pyszczynski et al., 2010)—and positive psychology—an approach that seeks to promote human flourishing (Seligman and Csikszentmihalyi, 2000). Existential psychological approaches are thoroughly invested in helping people come to terms with basic facts about life (e.g., mortality, isolation, freedom, meaning) and addressing potential anxiety that arises from contemplating such realities (Yalom, 1980). Positive psychological approaches focus on human strengths and what constitutes the good life, such as experiencing growth or reaching one's potential (Peterson, 2006). These two approaches have been largely treated as separate theoretical perspectives, until some recent critical work integrating the two (Wong, 2009). We agree with Wong (2020) and colleagues who see a fruitful synergy between these two fields and propose a shift toward existential-positive psychology. We advance that work by introducing a model that applies existential-positive psychology to the experience of human suffering.

In our view, any approach to human suffering must account for both the darker and lighter side of the human experience, and we posit that one must fully address the harsh existential realities that each person must address while simultaneously working toward sustainable flourishing for individuals, groups, and societies. Our theoretical model is based on prior work at the intersection of basic science and clinical practice that is more fully elaborated in Van Tongeren and Showalter Van Tongeren (2020). For a richer and more complete explication of our ideas, we encourage readers to start with that resource. In the present article, we formally introduce this model and its three core propositions and more fully explicate a similar existential positive psychology approach toward suffering in the midst of a global pandemic.

### Proposition #1: Suffering Elicits Existential Anxiety

The EPPMS views suffering as an existential issue. As we originally proposed (Van Tongeren and Showalter Van Tongeren, 2020), suffering is (a) *cognitively threatening* (i.e., it violates deeply held assumptions about the world), (b) *chronic* (persistent or enduring), and (c) *consequential* (i.e., it alters people in fundamental or profound ways). This differs from physical or mental pain, which is generally explainable, has a relatively short duration, and focused in one domain of life. These features of suffering—its cognitive threat, chronicity, and consequentialism—often reveal existential concerns that have the potential to generate considerable anxiety.

People have developed schemas, or working models, for how they organize, interpret, and make sense of the world (Taylor and Crocker, 1981). These schemata are embedded in cultures and often develop into consensually validated cultural worldviews, which are designed to provide people with meaningful explanations about the nature of life and humanity's place in it (Ibrahim, 1991). Cultural worldviews facilitate effective processing of social information and the navigation of social relationships, but they also help imbue one's life with meaning and manage potential existential anxiety (i.e., fear and dread regarding existential realities; Pyszczynski et al., 2010). For example, people may believe that life is fair and that good people receive what is fairly due to them; this belief in a just world helps people make sense of their life and the world around them (Lerner, 1980). Although this is a delusion that is largely inaccurate, such positive illusions have been found to be beneficial for coping with stress and trauma (Taylor, 1983; Taylor et al., 2000). Suffering often violates these longstanding beliefs (e.g., “I am a good person and good things happen to me because life is fair”) and shatter people's assumptions (Janoff-Bulman, 2010). When one's cultural worldview is rendered ineffective, they suddenly bear the full weight of existential anxiety (Vail et al., 2019). Put differently, cultural worldviews answer existential questions, and suffering undermines these safety-providing frameworks, leaving people prone to new questions, and fresh anxiety, resulting from contemplating existential realities. Indeed, suffering is an existential issue, as they must confront their core existential fears head-on.

Previous work has contended that there are five existential concerns (Koole et al., 2006). First, the world is chaotic and groundless, lacking an inherent structure; however, people must make decisions, and bear responsibility for such choices, amid myriad options. This gives rises to the burden of personal *freedom* (also known as groundlessness; Yalom, 1980): the weight of deciding one's future, often at the cost of foreclosing other options, all while facing external pressures on one's motivations (Iyengar and Lepper, 2000). Within this boundless world, each person's phenomenological experience is unique, rendering each person fundamentally separate from others and ultimately alone, resulting in a fear of *isolation* (Williams, 2007). Often, experiencing rejection or exclusion can impair psychological functioning, such as self-regulation (Baumeister et al., 2005) and prosociality (Twenge et al., 2007). Furthermore, each person may attempt to create a coherent narrative about their life and consistent identity that accounts for a wide range for experiences, which makes *identity* another pressing concern (McAdams and McLean, 2013). Although stress might make navigating concerns about identity more salient, other developmental points (e.g., adolescence, midlife) might also give rise to a shifting identity and concomitant existential anxiety. Despite the uncertainty of these three existential concerns, perhaps the only certainty in life is *death*: each human is keenly aware that they are mortal and, like every other animal, will someday perish (Pyszczynski et al., 2015). This realization of death elicits the potential for considerable existential dread and motivates a host of anxiety-attenuating behaviors, such as seeking self-esteem and defending one's cultural worldview (Solomon et al., 1991). Finally, the realization that one lives in a world that is formless but demands (often irrevocable) choices, in which one is isolated and must find an authentic identity, and in which the only certainty in life is that it will eventually end with death, one is likely to experience the final existential concerns rather acutely: *meaninglessness*. Meaninglessness is often viewed as a culmination of the other existential concerns (Yalom, 1980) and is a central feature of human life (Heintzelman and King, 2014) that is automatically defended (Van Tongeren and Green, 2010).

Suffering exposes all of these existential concerns: it highlights the unpredictable nature of the world and the human predicament of having to make choices nonetheless; it can isolate the suffering from those who are not actively suffering; it challenges people's views of themselves and their narrative story of their life; it can elicit fears of death or may directly increase the likelihood that one might die sooner, and; it can render a vision of the world as uncaring, cold, and meaningless. And left unaddressed, these concerns can cause considerable anxiety. In our view, suffering is an assault of existential anxiety (Van Tongeren and Showalter Van Tongeren, 2020).

### Proposition #2: Existential Anxiety Impairs Meaning

Our second proposition is that because suffering elicits existential anxiety, this directly impairs one's sense of meaning. Meaning is comprised of coherence, significance, and purpose and is generally defined as the subjective felt experience that's one life makes sense, is valuable, and is oriented toward something larger [see George and Park (2016) and Martela and Steger (2016)]. Because suffering often feels senseless (challenging coherence), can cause people to question whether or not they matter (threatening significance), and might reveal the absurdity of life (undermining purpose), suffering cuts across all dimensions of meaning. The relationship between meaning and existential concerns (and comfort) is well-documented.

Several social psychological theories converge on the notion that meaning helps people assuage existential anxiety. Terror management theory (TMT; Greenberg et al., 1986) asserts that the unique human capacity of self-awareness, coupled with advanced intellectual ability that facilitates symbolic thought, creates the awareness of one's mortality that has the potential to generate substantial terror. In order to manage this existential anxiety, individuals construct and adhere to cultural worldviews that imbue their existence with meaning, significance, and permanence (Pyszczynski et al., 2015). Similarly, the Meaning Maintenance Model (MMM; Heine et al., 2006) posits that meaning is a central motivation: humans are innate meaning-makers who strive to find a sense of meaning through self-esteem, certainty, belonging, and symbolic immortality. These efforts should mollify existential angst.

A conceptual cornerstone of these approaches is that under threat, the psychological mechanisms responsible for procuring safety and equanimity should be recruited as a way of reducing anxiety (e.g., Solomon et al., 2004). For example, if after being reminded of death, individuals defend their cultural worldview and derogate those who hold opposing views, it implicates those beliefs as central to providing existential solace (Arndt et al., 1997). Indeed, when the potential for anxiety is eliminated, cultural worldview defense is reduced (Greenberg et al., 2003). This highlights how the psychological efforts implicated after threats are designed to defend against existential anxiety and provide comfort.

These theoretical approaches have help generate a line of experimental work that has revealed that when existential concerns are made salient (e.g., death reminders, threats to meaning), people respond defensively and strive to reassert a sense of meaning in life. Even though there are individual responses to existential anxiety (e.g., Vess et al., 2009), research has found that implicit threats to meaning result in strategic defensive efforts aimed at restoring meaning and regaining psychological equanimity that occurs beyond conscious awareness (Van Tongeren and Green, 2010). That is, the proclivity to defend meaning is rather automatic.

This compensatory reaffirmation process is also supported by research on adversity and trauma. Park's (2010) meaning making model postulates that stress or adversity often creates a discrepancy between the way people expect the world to be (i.e., global meaning) and their assessment or evaluation of a particular event (i.e., situational meaning). The degree of this discrepancy is directly related to the amount of distress someone experiences. This distress results in an impaired sense of meaning in life, which triggers meaning-making efforts. Concordant with experimental existential approaches, the basic premise of all of these perspectives is that *existential threats reduce meaning and motivate efforts to restore meaning*.

Recent empirical work has confirmed this proposition. Across three studies, Edwards and Van Tongeren (2020) found that participants assigned to recall a time of suffering reported poorer mental health and well-being precisely because such suffering impeded their ability to find meaning in life. Suffering elicits existential anxiety, and existential anxiety challenges people's ability to find meaning in life, which has cascading negative effects on mental health and well-being.

### Proposition #3: Cultivating Meaning Allays Suffering

Given, then, that suffering (a) is an existential issue that (b) impairs one's sense of meaning in life, which leads to mental health concerns and hampers personal flourishing, we further contend that (c) cultivating meaning is a key to reducing suffering (Van Tongeren and Showalter Van Tongeren, 2020). Working from the tripartite definition of meaning as coherence, significance, and purpose (George and Park, 2016; Martela and Steger, 2016), restoring meaning in these domains should reduce suffering by directly addressing existential concerns and mitigating suffering.

Freedom, or groundless, often challenges meaning by undermining one's sense of coherence and making salient the inherent lack of structure in the world and weighty responsibility one has in making consequential choices in a chaotic world with near limitless options. People often turn toward their schemas to make sense of the world and find coherence. Given their central role in social cognition, worldviews, as schemas, are notoriously difficult to change, as evidenced by a host of cognitive biases, such as confirmation bias, where people selectively attend to information that align with their preexisting beliefs and discount information that runs contrary to it (Nickerson, 1998). However, suffering can often challenge one's worldview so sharply that it results in shattered assumptions (Janoff-Bulman, 2010) and requires a reorganization of one's worldview (Van Tongeren and Showalter Van Tongeren, 2020). This process is challenging, and often psychologically distressing; however, it can be a substantively powerful catalyst for personal growth and change (Yalom, 2008). Rebuilding meaning by honestly naming, accepting, and metabolizing one's existential frailty is key to experiencing a paradigmatic shift in one's understanding of the world that can lead to growth. Doing so helps people regain a sense of coherence.

Isolation often results in people feeling disconnected from others, which puts pressure on their sense of significance. Following social rejection, people often feel as though they do not matter (Stillman et al., 2009). However, given the fundamental need to belong, and the negative effects of exclusion on mental health (Morgan et al., 2007), excluded individuals strongly desire to reconnect (DeWall and Richman, 2011). Finding meaning through reestablishing a sense of relational connection can help reduce suffering. Relationships are often a primary source of meaning in life, and when people reengage in social relations with other, such as through relational repair behaviors as forgiveness, people report greater meaning in life (Van Tongeren et al., 2015). To the extent that people can restore a sense of significance and regain a feeling that they matter to other people in their life, their feelings of suffering should be allayed.

Identity concerns can leave people prone to feeling as though they have a disorganized sense of self and lack of a coherent narrative of their life (McAdams and McLean, 2013). However, when people are able to find meaning through developing a rich narrative that includes their suffering, the deleterious effects of such adversity begins to subside (Van Tongeren and Showalter Van Tongeren, 2020). This process often involves a reworking of the personal narrative, which can be facilitated by such approaches as narrative therapy (McAdams and Janis, 2004). Whether working with a professional or attempting to find a sense of identity on their own, people can build meaning through a coherent narrative, which, in turn, grants them with a sense of purpose in life.

Death can often pressure all of the dimensions of meaning by highlighting that the world does not make sense (i.e., challenging coherence), death indiscriminately comes us all (i.e., upending significance), and makes life finite and feel absurd [i.e., undermining purpose; see Van Tongeren and Showalter Van Tongeren (2020)]. Even still, there are ways to build meaning in light death reminders. First, people often invest in beliefs in their own symbolic immortality (i.e., that one's achievements will live on) as meaning-affirming strategies following threat (Van Tongeren and Green, 2010). Similarly, beliefs in literal immortality (i.e., religious beliefs in the afterlife) have been found to reduce death-related anxiety precisely because they make this life more meaningful (Van Tongeren et al., 2017). In addition, imbuing death with meaning facilitates terror management processes, such as reduced death-thought accessibility (Van Tongeren and Green, 2018). Indeed, affirmations of meaning are a way to address death and reduce suffering. Moreover, meaning is a central feature of human flourishing (VanderWeele, 2017). By building meaning across all domains should lead people to a fuller, richer, and more healthy life.

### Toward Flourishing

The centerpiece of our EPPMS is that meaning is a primary pathway toward wholeness, health, and flourishing in the midst of suffering. Although suffering makes it difficult to find meaning, which leads to numerous mental health effects [see Edwards and Van Tongeren (2020)], building sustainable meaning is paradoxically a salve for suffering (Van Tongeren and Showalter Van Tongeren, 2020). By focusing on the development of restoring and rebuilding meaning in the midst of suffering, people can begin to experience the potential for growth and flourishing. We also acknowledge that although our presentation of the propositions implies a certain linearity of progression, these constructs are likely reciprocally related and mutually reinforcing. Moreover, there are likely numerous moderators—such as content of one's worldview beliefs and the style with which one holds such beliefs, individual differences in personality, religious and spiritual orientation, and socioeconomic factors—that may alter one's experience of suffering and flourishing. These dynamics are complex. We encourage future research to address such nuances to advance research in this area (see Discussion).

The EPPMS asserts that the acceptance and engagement of existential realities, and the cultivation of meaning, is precisely what is necessary for people to metabolize experiences of suffering in ways that can (but do not necessarily) lead to transformative expressions of the good life. We draw from three relevant areas of empirical research that all share a common feature of meaning—transcending oneself. Accordingly, we see relationships (i.e., connecting with others), spirituality (i.e., connecting with the divine), and prosociality (i.e., improving the lives of others) as three chief candidates for ways that building meaning that addresses existential concerns can lead toward flourishing.

First, relationships are a centerpiece of meaning (Klinger, 1977). Building meaning through developing healthy relationships can not only help overcome the existential anxiety of concerns, such as isolation and death (Florian et al., 2002), but it can move people toward states of flourishing and wholeness (VanderWeele, 2017). Relationships are often a core contributor to well-being (Dush and Amato, 2005), and so by cultivating meaning via social interactions, people are simultaneously attending to their existential concerns and building a flourishing life of well-being and belonging.

Second, spirituality is a core feature of meaning (Wong, 1998) and plays an important role in managing existential terror (Vail et al., 2010). Indeed, there are different ways of being religious, of which some are more effective in reducing existential anxiety (Van Tongeren et al., 2016a), though religion/spirituality is central part of well-being and flourishing (Myers, 2008), especially later in life (Koenig et al., 1988). When people build meaning through connections to religion and spirituality (Park, 2005), they address the stress of existential concerns and transcend themselves to live richer and more flourishing lives.

Finally, prosociality has been a consistent source of meaning in life (Van Tongeren et al., 2016b). Not only does prosociality help reduce existential anxiety in the light of reminders of one's frailty (Jonas et al., 2002), but a recent meta-analysis confirms that it also contributes to well-being and flourishing life (Hui et al., 2020). In fact, experimental work has demonstrated the focusing on others, compared to focusing on oneself, enhances psychological flourishing (Nelson et al., 2016), providing additional evidence for the transformative nature of prosociality. Here, too, we see overlap between processes aimed at addressing existential concerns and building a flourishing life.

## Applying the EPPMS Framework to the COVID-19 Pandemic

To help demonstrate the theoretical and clinical utility of the EPPMS, we apply the propositions of the model to the COVID-19 global pandemic. First, both individually and collectively, the COVID-19 pandemic has revealed the five core existential concerns and elicited significant anxiety. An obvious candidate is *isolation*, given that many governments instituted weeks-long lockdowns at the beginning of the pandemic, and best scientific practices include social (physical) distancing from others as a way to mitigate the spread of the virus. Humans were designed to be connection with others (Baumeister and Leary, 1995), and the narrowing of social circles has caused distress for many. Indeed, recent longitudinal evidence has found that well-being attributed to social relationships has decreased due to the pandemic (VanderWeele et al., 2020). In fact, we see that for some, this need to belong has overridden the rational compliance for scientific advice, and people have continued to engage in close social interactions with others, often leading to increased spread of the virus.^1^

Related to this, many people have struggled with having many of their normal routines and structures disrupted, and the anxiety that comes from having to make difficult decisions about how to navigate life amid conflicting (or confusing) information about the virus. Thus, for many, the realization of existential *freedom* has been troubling; decisions about whether to travel, which family members or friends are safe, and how to conduct life and work amid vast uncertainty has been fatiguing and overwhelming for many. Many have felt conflicted and unsure of how to navigate an unpredictable world where their decisions have significant consequences for themselves and the lives of others.

This massive shift in daily life has also cause many people to wrestle with their *identity*. For those who defined themselves (at least in part) through their work, losing a job could have felt threatening both economically and existentially; those who took pride in their sociability may have felt as though a part of themselves is withering by not being around others; and the hobbies that might have helped define one's self-concept (e.g., being an athlete) may no longer be available (e.g., races were canceled or turned into individual virtual events). Some may have asked themselves who they are now, in the midst of this pandemic, when previously they would have defined themselves largely by their activities, employment, or hobbies.

Of course, this pandemic has also reminded us all of our mortality, as it has made *death* salient. As the worldwide death toll surges past 2 million, many know people whose lives have been claimed by this virus. This pandemic has revealed the frailty of human life and made clear that we shall all eventually die, which has led to increased reliance on cultural worldviews designed to mitigate such anxiety, such as increasing political polarization in the United States (Pyszczynski et al., in press). Still others might downplay the potential deadliness of the virus as a way of denying their own vulnerability and mortality [see Becker (1973)]. To be sure, a widespread reminder of human frailty is on full display in the midst of this pandemic.

This has led to a collective calamity for meaning—we suspect that there is widespread *meaninglessness* among many. Given the lengthy isolation, weightiness of freedom, challenges to identity, and reminders of death, people can struggle to find coherence, feel significant, or have purpose, leaving them with significant challenges to their own meaning. Thus, COVID-19 challenges many individuals' primary pathways to finding meaning. This pandemic has felt senseless for so many, as they struggle to come to terms with a seemingly random shared trauma and grief. Many have no schema for this once-in-a-generation event. It challenges feelings that they matter. And the disruption of daily social life has challenged what many to find a concrete and reliable purpose. A recent longitudinal study revealed that meaning in life significantly decreased from pre-pandemic (January, 2020) to mid-pandemic (June, 2020), supporting the claim that meaning has been impaired by the suffering elicited by the COVID-19 pandemic (VanderWeele et al., 2020). We have yet to see the downstream effects of lost meaning on mental health and social functioning.

So how can people respond to the pandemic in ways that might facilitate flourishing? Drawing from the EPPMS, we contend that cultivating meaning in the midst of this ongoing pandemic is a primary way to address these concerns and their concomitant anxiety, and doing so operates as a protective factor for mental health and can lead to human flourishing. Indeed, recent research has supported this notion directly: greater meaning in life is associated with less anxiety and stress amidst the COVID-19 pandemic (Trzebiński et al., 2020). People who are able to find meaning report better psychological health and well-being (Hooker et al., 2020). Thus, we suspect that building meaning will help those suffering in the midst of this pandemic fare better in the long term.

## Clinical Implications of the EPPMS

The EPPMS lies at the intersection of theory and practice. It is an empirical model with considerable research support, and it is also a clinically relevant model that can practitioners engage with their clients who are suffering. To advance the principles of the EPPMS in work with clients, we pay special attention to integrating the theoretical assertions of the EPPMS in clinical work. We highlight the flexibility of the EPPMS and how it applies to work with clients who are suffering from a wide range of concerns, from personal issues to global crises.

The EPPMS contends that suffering is an existential issue; that is, it makes existential realities salient, which can lead to increased existential anxiety. To be sure, myriad clinical approaches have long addressed the concept of suffering. However, many describe suffering as a “state of mind.” That is, some approaches argue that suffering is avoidable, largely a result of cognitive distortions, irrational thoughts, or a misalignment between cognitions and emotions. On the contrary, the EPPMS posits that because suffering raises existential concerns, and each person must face these realities and their potential anxiety, suffering is a certainty in life—each person will suffer at some point in life (Van Tongeren and Showalter Van Tongeren, 2020). Rather than pathologize suffering as an inherently problematic result of a client's cognitive or affective processing, the EPPMS asserts suffering is a normal, human experience. For example, a terminal illness is likely to raise existential concerns and cause suffering, and no amount of cognitive gymnastics, thought correction, or emotional realignment can deny the reality that one is going to die. Instead, the EPPMS is focused on two primary clinical goals: *existential acceptance* and *cultivating meaning*. We explicate these below.

### Clinical Goal 1: Existential Acceptance

The first two propositions of the EPPMS are that (a) suffering elicits existential anxiety, and (b) existential anxiety can impair meaning. When faced with such anxiety, people often respond defensively [see Hayes et al. (2010)]. Indeed, some people respond by reducing self-awareness (Arndt et al., 1998) or avoiding existential considerations altogether (Bond et al., 2011). When people disengage from themselves and the realities of the world, it can lead to greater distress (Van Tongeren and Showalter Van Tongeren, 2020). For that reason, the first clinical strategy is to help clients to move toward acceptance—of their suffering and of the existential realities their suffering reveal.

How might therapists help facilitate this? First, clinicians can engage in psychoeducation regarding the five existential concerns. Talking openly and honestly about what these five concerns are and how these realities are common questions that each person faces helps destigmatize their experience and can situate their suffering in a broader context. Therapists can approach suffering like they may approach some trauma work: to help clients understand what it feels like in their body when they experience existential fears (Van der Kolk, 2015). In addition, this work can help clients gain a sense of personal autonomy wherein they gain some control regarding strategies to use to buffer this anxiety.

In the case of the COVID-19 pandemic, a clinician might help a client realize that feelings of persistent groundlessness and the weight of *freedom* may lead to anxiety and decision fatigue. Some initial psychoeducation can reframe their experience as a common feature of being human and having to make certain decisions in an uncertain and chaotic world. Or, perhaps a client is reporting feeling hopeless and depressed because they feel as though they have lost their sense of self, being disconnected from things that gave them purpose. Someone who identified as a traveler or athlete may struggle when travel is restricted and events are canceled. Working to help clients identify that such anxiety is a result of concerns surrounding *identity* can impel clinical work toward accepting this new reality. As long as the client ignores or denies the reality of their suffering, they will be unable to move forward toward flourishing. However, after acceptance, clients struggling with identity-related concerns might work toward crafting a new narrative in light of their current reality (e.g., exploring their current town or state; finding new ways to exercise or compete). However, deep, lasting therapeutic work on existential issues is not possible until people begin to acknowledge and accept their suffering as an existential reality.

### Clinical Goal 2: Building Meaning

Following acceptance, clinicians can work collaboratively with clients to developing lasting strategies of cultivating meaning. Previous work has highlighted the importance of therapeutic efforts aimed to enhance meaning (Wong, 2010), and a meta-analysis of clinical work found that meaning-focused therapy was more successful than standard treatments, precisely because of the meaning provided by such approaches (Vos, 2016). This positive effect of such approaches has been found to improve health (Roepke et al., 2014), including among cancer survivors (Canada et al., 2016). But what might building meaning look like across the five concerns?

Clients with concerns about *freedom/groundlessness* might need some help reorganizing their worldviews that have been shattered by suffering. Because worldviews provide structure, and suffering can pressure the assumptions that many people hold about themselves and the world, those who are feeling groundless or anxious about freedom may need support working through the deconstruction and reconstruction of some beliefs, or perhaps larger parts of their worldviews (Van Tongeren and Showalter Van Tongeren, 2020). Finding an authentic set of beliefs that better reflects their suffering can enhance meaning, largely by providing coherence.

When seeing clients whose concerns center on *isolation*, two parallel approaches can be adopted. First, clinicians can work with the client to develop self-talk that is comforting, validating, and loving. Cultivating self-compassion is an important feature of well-being (Zessin et al., 2015), and one that can be facilitated when people are truly distant or disconnected from others. Second, clinicians can attempt to help their clients develop healthy and mutual relationships that can meet some of their fundamental belonging needs (Baumeister and Leary, 1995). Helping clients understand that we truly are isolated but can find transcendent moments of connection with others can be powerful. For example, research on I-sharing has revealed how people can find deep connections with others, and it is a practice that can modeled in the therapy office (Pinel et al., 2015). When people can find connections, with themselves or other people, they begin to find meaning through feeling significant and as though they matter.

Those who are struggling with *identity* may need help forming a new narrative that (a) includes their suffering, and (b) reflects their new reality, as it may have changed due to their suffering. Acknowledging, and owning, their struggles as part of their own story can be liberating and empowering. This narrative work seeks to integrate both who they saw themselves as before their suffering and how they view themselves as a result of their suffering, with the goal being an integrated sense of self. Another benefit of narrative work can be finding meaning (McAdams and Janis, 2004), such as a sense of purpose.

Concerns about *death* are common, and reminders of our mortality can be unsettling. But there are ways to build meaning under the weighty reality our human finitude. One such therapeutic technique is to have clients write their own obituary. Asking clients what they want to be remembered for (i.e., symbolic immortality) can help them reprioritize their values and reorient their life toward behaviors that are consistent with these goals. For example, a client who wants to be remembered as generous can take concrete steps of generosity to build meaning [see Van Tongeren et al. (2016b)]. Indeed, a recent review of research has found the multifarious ways in which death awareness can lead to positive trajectories (Vail et al., 2012), each of which may contribute to a sense that life is meaningful.

Addressing each of these concerns may help build a strong sense of meaning; however, some clients may still report a nagging sense of *meaninglessness*. After revealing the existential realities of freedom, isolation, identity, and death, some clients may fall into a nihilistic depression, in which they embrace the absurdity of life and contend that nothing in life is meaningful. However, persistent nihilism is not tenable. The paradox of nihilism is that it, too, is a meaning system. It provides a sense of coherence that helps people make sense of the (senseless) world. Some have argued that the harsh confrontation with existential realities is *precisely* what is needed to jostle people into radical periods of growth and transformation (Yalom, 2008). We affirm that humans are natural meaning-makers (e.g., Heine et al., 2006), and part of the therapeutic work is to help clients understand how they make meaning, so that they can bring it into their conscious awareness. Doing so will help them develop coherence, feel significant, and derive lives of purpose that permeate their daily experiences.

## Novelty of the EPPMS

This model is based on the merging of two lines of theoretical and empirical inquiry: existential psychology and positive psychology. Accordingly, and as noted above, we draw from previous empirical research on existential psychology, such as TMT (Pyszczynski et al., 2010, 2015) and the MMM (Heine et al., 2006; Van Tongeren and Green, 2010), and conceptual work from Yalom (1980, 2008) and Wong (2010) on the importance of existential themes in human functioning and clinical practice. We also integrate positive psychology work (Peterson, 2006; Park, 2010; Heintzelman and King, 2014; George and Park, 2016; Hooker et al., 2020), as we advocate for an existential-positive psychology approach to suffering (Wong, 2009, 2020). As such, our model is decidedly influenced by many intertwining conceptual perspectives and bodies of empirical work [see Van Tongeren and Showalter Van Tongeren (2020) for a full review].

Still, our model makes several novel contributions. First, we make suffering as an existential issue as a centerpiece of our model. In our view, experiences of suffering give rise to existential anxiety that erodes meaning and impairs psychological functioning. The emphasis on how suffering is existential, and the importance of addressing the underlying existential concerns when clients report distress, provides a different vantage point for engaging with suffering, both empirically and clinically. We see this novel entry to research and practice as valuable.

Second, we contend that building meaning is the key to flourishing when experiencing suffering. Other approaches, such as the medical model, view suffering and flourishing at odds. We disagree with this false dualism. Rather, as evidenced from individuals living with chronic conditions or terminal diagnoses, people may experience considerable flourishing while suffering. Suffering does not need to be eliminated in order for people to thrive; rather cultivating meaning amid such circumstances improves one's well-being. This assertion stands in stark contrast to many other existing approaches.

Third, we depart from other positive psychology approaches that tend to focus more on happiness or hedonic well-being. Such subjectively positive affective states may feel elusive amid suffering, whereas meaning can be cultivated in stress and hardship (Baumeister et al., 2013). Rather, we focus on meaning, conceptualized as eudemonic well-being marked by coherence, significance, and purpose. This inclusion of both the “light” and “dark” side of human nature, as well as the realization that positive psychology could benefit from expansion to considering a broader set of psychological states, is aligned with Positive Psychology 2.0.

Finally, and perhaps most crucially, our model identifies the mechanism by which suffering can lead toward flourishing (or perhaps perceived growth): cultivating meaning. By pinpointing the process through which suffering can be metabolized, we provide an empirically testable hypothesis and a clinically tangible application for those who are suffering. Initial empirical work in this area is promising [see Edwards and Van Tongeren (2020)], and meta-analytic results suggest building meaning in clinical settings is powerfully effective (Vos, 2016). Still, we see more work to do to catalyze work in this area.

## Advancing the EPPMS

We have proposed a new model of and existential positive psychology of suffering, termed the EPPMS, and have provided research support and clinical implications of this model. However, we see several fruitful areas for future research inquiry. Below, we discuss possible avenues forward, as well as some open questions.

### Providing Additional Direct Support for the Propositions of the Model

The EPPMS was based on considerable research. However, to date, little research has focused on testing the propositions in this model. A notable exception is Edwards and Van Tongeren (2020), who reported three studies in which participants who were randomly assigned to recall suffering reported lower meaning in life, which, in turn, was associated with poor mental health. Thus, this provided direct support for the EPPMS and the mediating role of meaning in life in the association between suffering and distress. Still, much more work is needed to test each of these three theoretical propositions of the model, as well as potential moderators and contextual factors. We encourage that such work, to the fullest extent possible, should employ experimental or longitudinal designs to help establish causal effects and the temporal ordering of the central variables of interest. The empirical work on which we based this conceptual was conducted both on general populations [e.g., TMT work; see Pyszczynski et al. (2015)] and with community members undergoing acute trauma and suffering [e.g., see Park (2010) for a review]. In addition, many measures were subjective self-reports. Accordingly, we also encourage future endeavors to draw upon multiple populations, including samples recruited specifically for their experiences of suffering (e.g., Canada et al., 2016), using a range of methodological assessments (e.g., behavioral indicators, objective measures, physiological responses, biomarkers) to provide additional evidence for the validity of our proposed model.

### Integrating Basic and Applied Science

In a related vein, much, but not all, of the research reviewed has been conducted in laboratory settings, where highly controlled settings can offer internal validity and increase confidence in the causal factors under investigation [see Pyszczynski et al. (2010) for a review]. However, much more work is needed in real clinics or private practice settings. We strongly encourage a greater connection between application and research. For example, researchers could partner with practitioners to sample individuals reporting existential concerns in their therapy sessions [see Pinel et al. (2015)]. Alternatively, larger data collection efforts, such as a nationally representative sample of individuals who report existential suffering, could reveal individual differences in the ability to find meaning in life and work toward flourishing; cross-cultural work could identify those features that rather universal and those that are culturally-specific [e.g., see VanderWeele (2017) and VanderWeele et al. (2020)].

### Shifting Clinical Perspectives

Elsewhere, we have offered a fuller account for an existential-positive psychology approach to suffering [see Van Tongeren and Showalter Van Tongeren (2020)]. We encourage a broader shift in how clinicians view therapy. Currently, the predominance of the medical model and pressures from insurance companies impel clinicians to “fix” clients and “reduce symptoms,” often through cognitive behavioral therapy (CBT) techniques. However, CBT is not effective when there is no cognitive distortion; a terminally ill client whose cognitions include “I am going to die” is not thinking irrationally. The pressure clients and clinicians feel under the medical model to “restore” clients back to baseline functioning creates undo suffering and ignores the tenable reality that suffering changes how people perceive the world around them. It is a faulty premise that someone who has experienced suffering would not be changed by the suffering in and of itself. Rather, looking at suffering from an existential positive psychology perspective, where the reality informs how one might live with their remaining time, moves from an “irrationality” perspective to a strengths-based perspective, where they desire to build meaning and experience flourishing. This could help inform practice and give clinicians the tools to help work with clients who are experiencing increasingly difficult times. This likely also requires that clinicians engage in their own existential work to examine and understand how their clients' suffering has affected their own existential worldviews. We argue that helping clinicians build existential resilience might also be able to reduce burnout.

### Developing Interventions for Enhancing Existential Resilience

A fundamental assumption of most existential approaches is that encountering these realities can lead to considerable anxiety. However, shifting toward a perspective more strongly informed by positive psychology, we suspect that humans should be able to develop *existential resilience*, or the ability to encounter existential concerns are truths and not threats, facts and not fears (Van Tongeren and Showalter Van Tongeren, 2020). We suspect that more work should be dedicated toward developing this construct and then developing interventions focused around existential resilience and persistent questions about what it means to be human. Doing so should be helpful to provide a durable buffer against the existential anxiety engendered by considering life's deepest realities.

## Concluding Remarks

We presented the EPPMS, which is an approach to suffering based on the intersection of existential and positive psychology, with the primary goal of alleviating suffering through the cultivation of meaning. Drawing from empirical research and clinical practice (Van Tongeren and Showalter Van Tongeren, 2020), we see this framework as a useful theoretical and clinical model for those who are experiencing indelible and profound struggles that raise fundamental questions about what it means to be human. The application of this model to collective suffering, such as the COVID-19 pandemic, demonstrates its utility and generalizability. We hope that future research will continue to advance work in this exciting area of inquiry and practice.

## Data Availability Statement

The original contributions presented in the study are included in the article/supplementary material, further inquiries can be directed to the corresponding author/s.

## Author Contributions

DV and SS developed the model and wrote the manuscript. Both authors contributed to the article and approved the submitted version.

## Conflict of Interest

The authors declare that the research was conducted in the absence of any commercial or financial relationships that could be construed as a potential conflict of interest.

## Footnotes

^1^We acknowledge that there are multiple reasons why people may ignore scientific advice, including disbelief of or mistrust of science. However, we suspect that the need to belong accounts for some of the motivation.
